# “If We Can Get Them to Stop, They Can Have Such a Better Life”: Implementing Tobacco and Nicotine Dependence Treatment Services in Community Pharmacies in North Dakota

**DOI:** 10.5888/pcd22.250088

**Published:** 2025-07-17

**Authors:** Hailey M. Wanner, Kelly Corr

**Affiliations:** 1 North Dakota State University, Fargo

Although cigarette use among high school students and adults has declined since its peak in 1997, in North Dakota, nearly 1 in 3 high school students instead use e-cigarettes, and approximately 1 in 5 adults continue to smoke (1). The prevalence of tobacco and nicotine dependence poses substantial public health challenges, especially in rural communities (2).

More than 480,000 people, equivalent to the average capacity of 8 professional football stadiums, die from cigarette smoking annually in the US (3). In North Dakota, 1,000 adult deaths annually are attributed to cigarette use (1). Of cancer-related deaths in North Dakota, approximately 1 in 4 are associated with smoking (1). Cigarette use results in a high economic burden: in 2018, it cost the US more than $600 billion, including $240 billion in health care spending and nearly $185 billion in lost productivity due to smoking-related illnesses and health conditions (4). In 2021, health care expenditures attributed to tobacco use in North Dakota totaled $326 million, approximately equivalent to spending $421 for each person living in the state that year (1). Annual smoking-related lost productivity equates to nearly $185 billion in the US and $233 million in North Dakota (1,4). It is clear why the Centers for Disease Control and Prevention cites cigarette smoking as the leading cause of preventable disease, disability, and death in the US (3).

Smoking is a behavior that can harm nearly every organ in the human body, increasing the risk of heart disease, stroke, lung disease, diabetes, and cancer, and resulting in a substantial impact on population health (3). This essay explores and promotes providing tobacco and nicotine dependence treatment in the community pharmacy setting to increase patient care opportunities and improve health outcomes, particularly in rural areas.

## The Profession of Pharmacy

Pharmacists are highly accessible and trusted health care professionals (5). Community pharmacies are a key component of the health care system, especially in rural, medically underserved areas, and they present an opportunity to help people quit using tobacco and nicotine products (5). Our ethnographic graduate research focuses on piloting an education-based intervention to assist independent community pharmacies in North Dakota in addressing tobacco and nicotine use among their clients. Our preliminary research results support the concept that in smaller communities, people often have close relationships with each other, including their local pharmacist. In one of our research pilot sites, a pharmacy in a rural town, a staff pharmacist said, “We care about our patients, and we want the best for their health.” To expand their scope of practice and fill gaps in access to health care services, independent community pharmacies are increasingly offering clinical services and improving patient outcomes (6).

## Tobacco and Nicotine Dependence Treatment

Smoking cessation, the process of quitting the use of cigarettes, is more formally called tobacco dependence treatment (7). To encompass cigarette use as well as use of other tobacco or nicotine products, we use the term “tobacco and nicotine dependence treatment.” The main components of this treatment are behavioral therapies and medications. Among the behavioral therapy options are cognitive behavioral therapy, motivational interviewing, mindfulness practices, and support from technology-based options such as telephone quitlines, text message communications, or online media platforms (7). Nicotine replacement therapy (NRT) products are offered in various formulations, including patches, gum, lozenges, and nasal spray. All NRT products are deemed equally effective and are estimated to increase treatment success by 50% to 70% (7). Multiple NRT products can be used concurrently and are thought to provide better relief of withdrawal symptoms and cravings (7). The US Food and Drug Administration (FDA) has approved bupropion and varenicline as oral tobacco cessation medications. Bupropion and NRT have been shown to be equally effective, and some studies suggest varenicline is more effective than bupropion alone or the use of a single form of NRT (7). Bupropion and varenicline can be used in combination with NRT, which allows prescribers to tailor a person’s tobacco and nicotine dependence treatment plan to their individual needs (7).

## Implementing Tobacco and Nicotine Dependence Treatment in Community Pharmacies

The implementation of tobacco and nicotine dependence treatment in community pharmacies can bolster the clinical capabilities and public health impact of community pharmacies. As of March 2025, eighteen states had implemented legislation allowing pharmacists prescriptive authority to provide patients with tobacco and nicotine dependence treatment medications (8). Of these, 9 states allow pharmacists to prescribe all medications approved by the FDA for smoking cessation, and the other 9 allow NRT only (8). In 2021, pharmacists in North Dakota were granted the authority to independently prescribe all FDA-approved medications, including varenicline, bupropion, and NRT (9). In the following year, the state’s Medicaid program expanded their coverage to include tobacco and nicotine dependence counseling provided by pharmacists (10). This expanded coverage broadened the impact of pharmacists on the adult Medicaid population in North Dakota, whose prevalence of smoking is more than double the prevalence among all adults in the state (39.1% vs 17.4%) (10).

Other insurers permit pharmacists to become recognized as medical providers, which allows them to submit reimbursement claims for tobacco and nicotine dependence treatment consultations as well as for the medications and NRT products they prescribe (5). These additional incentives may increase the number of encounters between pharmacists and people who smoke and lead to a reduction in cigarette use. During an unstructured interview conducted as part of our ethnographic graduate research, a pharmacist offering tobacco and nicotine dependence treatment services said, “These people have control over it [their tobacco and nicotine use]. If we can get them to stop, they can have such a better life. I honestly . . . I feel very strongly about this.”

Some independently owned community pharmacies in North Dakota have become pioneers in offering tobacco and nicotine dependence treatment to their patients. They use Ask-Advise-Refer/Connect, a method that combines the approaches of Ask-Advise-Refer and Ask-Advise-Connect (11). Both approaches share the steps of engaging patients by asking about tobacco use and advising them to quit. The difference lies in what actions are taken in the last step. In Ask-Advise-Refer, the patient is given a referral to a resource for quitting, whereas in Ask-Advise-Connect, the patient is directly connected to a resource for quitting (11). A pharmacist using Ask-Advise-Refer/Connect can choose to make a referral or connect with the patient to provide treatment at the pharmacy, whichever the patient prefers (11). Referrals can be made to state quitlines or local public health units, which assist in providing behavioral counseling and free NRT products. Because pharmacists in North Dakota have the authority to prescribe tobacco and nicotine dependence treatment medications, patients who are ready to quit can be immediately connected to pharmacists and receive treatment at the pharmacy. Regardless of whether a patient is provided with a referral or a connection, the pharmacist should follow up with patients on their progress toward cessation during future pharmacy visits. The second author (K.C.) developed a flowchart describing how a patient progresses through a tobacco and nicotine dependence treatment support process (Figure).

**Figure:**
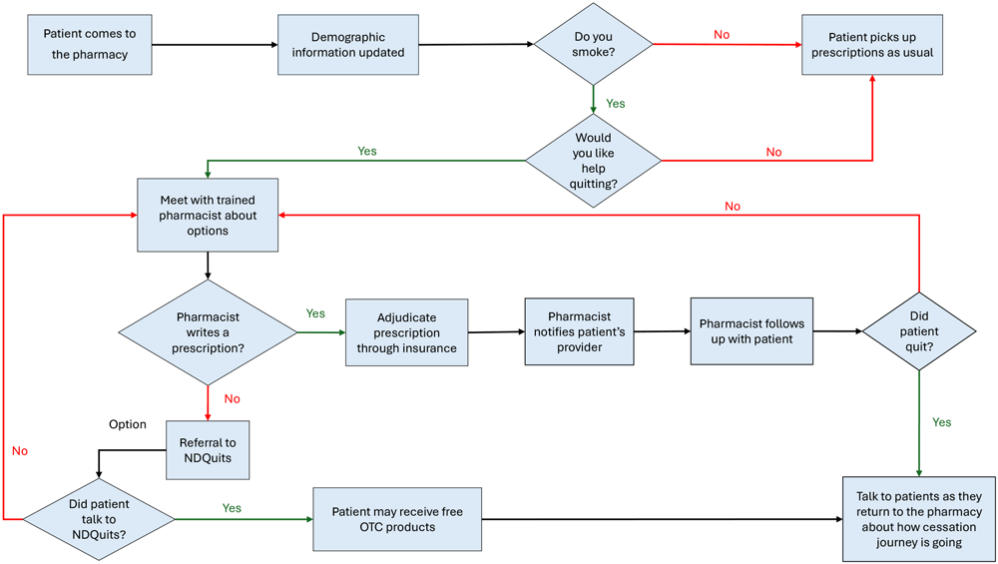
Basic pharmacy workflow for tobacco and nicotine dependence treatment in North Dakota. NDQuits is the state tobacco quitline. Over-the-counter (OTC) products refer to nicotine replacement products that can be acquired without a prescription.

## Call to Action

Pharmacists are called to be public health professionals and capitalize on opportunities to provide tobacco and nicotine dependence treatment for their patients, especially in rural areas. This expansion of services necessitates strengthening knowledge of tobacco and nicotine dependence treatment medications, learning how to provide behavioral counseling, and completing the requirements to be recognized as a provider of tobacco and nicotine dependence treatment services by health insurers.

The training of pharmacy students should be studied to ensure they can take the initiative to offer new services, apply population health strategies, and as a result, better serve their patients’ health care needs. Practicing pharmacists may need to refresh their knowledge and skills to provide tobacco and nicotine dependence treatment. Continuing education is a professional requirement, and pharmacists should actively seek opportunities to learn about topics such as motivational interviewing, tobacco and nicotine dependence treatment counseling, and current trends in tobacco use. In states where tobacco and nicotine dependence treatment provided by pharmacists is not yet authorized, pharmacists are encouraged to work with their board of pharmacy and local pharmacy organizations to advocate for expanding patients’ access to clinical services in community pharmacy settings.

Billions of dollars and hundreds of thousands of lives are lost to cigarette smoking every year in the US. Promoting pharmacy services and ensuring future pharmacists’ readiness for success should be a top priority for the profession. The next step toward preventing the disease, disability, and death attributable to tobacco use lies with pharmacists implementing tobacco and nicotine dependence treatment in community pharmacies across the country.
